# Pyrrolizidine alkaloid variation in *Senecio vulgaris* populations from native and invasive ranges

**DOI:** 10.7717/peerj.3686

**Published:** 2017-08-14

**Authors:** Dandan Cheng, Viet-Thang Nguyen, Noel Ndihokubwayo, Jiwen Ge, Patrick P.J. Mulder

**Affiliations:** 1 State Key Laboratory of Biogeology and Environmental Geology, China University of Geosciences (Wuhan), Wuhan, China; 2 School of Environmental Studies, China University of Geosciences (Wuhan), Wuhan, China; 3 Faculity of Biology and Agriculture Engineering, Thai Nguyen University of Education, Thai Nguyen, Vietnam; 4 Département des Sciences Naturelles, Ecole Normale Supérieure, Bujumbura, Burundi; 5 Hubei Key Laboratory of Wetland Evolution & Ecological Restoration, China University of Geosciences (Wuhan), Wuhan, China; 6 RIKILT, Wageningen University & Research, Wageningen, The Netherlands

**Keywords:** Secondary metabolite, Qualitative defense, Diversity, Biological invasion, Shift Defense Hypothesis (SDH), Liquid chromatography-tanderm mass spectrometry (LC-MS/MS)

## Abstract

Biological invasion is regarded as one of the greatest environmental problems facilitated by globalization. Some hypotheses about the invasive mechanisms of alien invasive plants consider the plant–herbivore interaction and the role of plant defense in this interaction. For example, the “Shift Defense Hypothesis” (SDH) argues that introduced plants evolve higher levels of qualitative defense chemicals and decreased levels of quantitative defense, as they are released of the selective pressures from specialist herbivores but still face attack from generalists. Common groundsel (*Senecio vulgaris*), originating from Europe, is a cosmopolitan invasive plant in temperate regions. As in other *Senecio* species, *S. vulgaris* contains pyrrolizidine alkaloids (PAs) as characteristic qualitative defense compounds. In this study, *S. vulgaris* plants originating from native and invasive ranges (Europe and China, respectively) were grown under identical conditions and harvested upon flowering. PA composition and concentration in shoot and root samples were determined using Liquid Chromatography-Tandem Mass Spectrometry (LC-MS/MS). We investigated the differences between native and invasive *S. vulgaris* populations with regard to quantitative and qualitative variation of PAs. We identified 20 PAs, among which senecionine, senecionine N-oxide, integerrimine N-oxide and seneciphylline N-oxide were dominant in the roots. In the shoots, in addition to the 4 PAs dominant in roots, retrorsine N-oxide, spartioidine N-oxide and 2 non-identified PAs were also prevalent. The roots possessed a lower PA diversity but a higher total PA concentration than the shoots. Most individual PAs as well as the total PA concentration were strongly positively correlated between the roots and shoots. Both native and invasive *S. vulgaris* populations shared the pattern described above. However, there was a slight trend indicating lower PA diversity and lower total PA concentration in invasive *S. vulgaris* populations than native populations, which is not consistent with the prediction of SDH.

## Introduction

An alien invasive plant species is a species that expands its natural range with facilitation from intentional or non-intentional human activities, tending to hazard biodiversity, ecosystem services and human well-being in its new range (Vilà & Hulme, 2017). Many hypotheses have been proposed to explain the invasive mechanisms of alien invasive plants (Catford, Jansson & Nilsson, 2009). Some explanations focus on plant-herbivore interactions and the role of plant defense. For instance, the “Enemy Release Hypothesis” (ERH) states that in a new range, introduced plants may leave behind their specialist herbivores and gain a rapid increase in distribution and abundance (Keane & Crawley, 2002). Loss of enemies leads to lower defense levels as plants allocate fewer resources to defense and more to growth, as according to the “Evolution of Increased Competitive Ability” (EICA) hypothesis (Blossey & Notzold, 1995). The “Shift Defense Hypothesis” (SDH) argues that invasive plants decrease the level of quantitative defense but increase their qualitative defense, as invasive plants still face pressure from generalist herbivores even though they escaped attack from specialists (Doorduin & Vrieling, 2011; Joshi & Vrieling, 2005; Müller-Scharer, Schaffner & Steinger, 2004).

Chemical defense in plants can be divided into qualitative defense and quantitative defense in relation to their effect on herbivores. Plant secondary metabolites (SMs) involved in qualitative defense are toxic to many herbivores and not very costly to produce. Those involved in quantitative defense are based on digestibility-reducing chemicals and more expensive to produce and to maintain due to the typically higher complexity of the molecules (Feeny, 1976; Rhoades & Cates, 1976). Specialist and generalist herbivores react in different ways to toxic SMs: generalist herbivores are deterred by high concentrations of toxic chemicals, while specialists are often adapted to these chemicals and use them as a cue to find their host plant. Thus, plants containing high concentrations of toxic chemicals suffer more from specialist herbivores (Cates, 1980). Hence, specialist and generalist herbivores inflict different selective pressures on plants, and the concentration of SMs is balanced by the opposing selective forces of specialists and generalists (“Specialist-Generalist Dilemma”, Van der Meijden, 1996).

Moreover, different plant metabolites, even from the same groups of chemicals, may have different effects on herbivores (Kleine & Mülller, 2010; Macel et al., 2005; Van Dam et al., 1995). It is assumed that plants with a more diverse and/or with higher concentrations of SMs can better protect themselves when the specialist herbivores adapted to the qualitative defense chemicals are absent. Therefore, for introduced plants variation in both concentration and composition of defense chemicals is important to defend themselves against the guild of herbivores in a new range.

*Senecio* and *Jacobaea*, possessing pyrrolizidine alkaloids (PAs) as their characteristic defense compounds, have been chosen in several studies as model species to assess the quantitative and qualitative variation in SMs in native and introduced populations. PAs act as deterrents or toxins to non-adapted herbivores and pathogens. However, specialist herbivores that are adapted to PAs can utilize them from host plants for their own benefit, such as for a food cue and oviposition (Joosten & Van Veen, 2011; Macel, 2011; Trigo, 2011). Higher concentrations of PAs have been found in invasive rather than native populations of *Jacobaea vulgaris* (syn. *Senecio jacobaea;* Joshi & Vrieling, 2005; Lin, Klinkhamer & Vrieling, 2015), and invasive *Senecio pterophorus* was found to have a higher concentration of PAs than its conspecific relatives (Caño et al., 2009; Castells, Mulder & Perez-Trujillo, 2014). Beside PAs from *Senecio* and *Jacobaea*, more than 350 PAs have been identified in an estimated 6,000 plants in the Boraginaceae, Asteraceae, and Leguminosae families (Stegelmeier et al., 1999). In this study, we selected *Senecio vulgaris* (common groundsel, Senecioneae: Asteraceae) as a model organism for the comparison of quantitative and qualitative PA variation between native and invasive populations. *S. vulgaris*, a cosmopolitan weed in temperate regions, probably originated from southern Europe (Kadereit, 1984), and has spread to America, North Africa, Asia, Australia and New Zealand in the 18th century (Robinson et al., 2003). The occurrence of *S. vulgaris* was first recorded in China in the 19th century, and it is nowadays mainly distributed in northeastern and southwestern China (Li & Xie, 2002; Xu et al., 2012). *S. vulgaris* plants of some European and Canadian populations contain high amounts (>0.6 mg/g fresh weight) of PAs (Von Borstel, Witte & Hartmann, 1989). Handley et al. (2008) investigated the invasive mechanisms of this species with respect to the interaction between plants and pathogens and the outcomes did not support the EICA hypothesis. Zhu et al. (2017) found that although *S. vulgari* s might have been introduced into China on multiple occasions, the Chinese populations contained smaller genetic diversity compared to European populations.

In this study, *S. vulgaris* plants from seeds collected from 6 native (Europe) and 6 invasive (China) populations were grown under identical conditions in a greenhouse. PAs were extracted from the roots and shoots of harvested *S. vulgaris* plants and measured using Liquid Chromatography-Tandem Mass Spectrometry (LC-MS/MS). According to the SDH, invasive plants tend to evolve higher levels of qualitative defense chemicals. Hence, we hypothesized that plants from invasive *S. vulgaris* populations would produce higher concentrations of PAs than those from native ranges. We also compared PA profiles in the native and invasive populations.

## Materials and Methods

### Studies species

*Senecio vulgaris* can complete its life cycle in as little as 8 weeks, producing an average of 38,300 seeds per generation and can be found in gardens, lawns, roadsides, field margins, arable lands, waste places and coastal habitats. Variation in capitula morphology, seed dormancy and growth form have been observed in different *S. vulgaris* populations (Robinson et al., 2003). No surveys have yet been undertaken on the amount of herbivory naturally occurring in *S. vulgaris* populations. However, it is known that *S. vulgaris* can be the host plant of generalist herbivores such as the leafminer *Liriomyza trifolii* and the Western tarnished plant bug (*Lygus hesperus*) (Minkenberg & Lenteren, 1986; Barlow, Godfrey & Norris, 1999). The cinnabar moth (*Tyria jacobaeae*), flea beetle (*Longitarsus jacobaeae*) and ragwort seed fly (*Botanophila seneciella*) are specialists that have be used as biological control for *Jacobaea vulgaris* in North America and Australia. The first two insects have been observed also to feed on *S. vulgaris*, but it is unknown whether the ragwort seed fly can feed on *S. vulgaris*. Furthermore, a rust fungi *Puccinia lagenophorae* can infect *S. vulgaris* plants and is used as biological control of *S. vulgaris* (Frantzen & Hatcher, 1997). In China, we observed that leafminers and seed flies caused damage to natural populations of *S. vulgaris* and we also observed heavy herbivory by aphids on *S. vulgaris* plants grown in the greenhouse for this study. The insects have not yet been identified, and it remains to be determined whether these are specialists or not.

Some *S. vulgaris* biotypes showed increased resistance to various herbicides such as simazine, atrazine, bromacil, pyrazon, buthidazole and linuron. Therefore, *S. vulgaris* is considered as a troublesome weed, especially in horticulture where frequent cultivation occurs (Robinson et al., 2003). The morphology of *S. vulgaris* plants resembles that of some other *Senecio* species used as Chinese traditional medicinal plants, implicating a risk to human health if they are used as medicine or otherwise consumed by mistake (Yang et al., 2011).

### Plant resources, growth and harvesting

We used seeds collected from 6 native and 6 invasive *S. vulgaris* populations in Europe and China (Table 1). Achenes from 6 to 20 individual plants per population were kept in paper bags, air-dried and stored in the laboratory. Seeds from 4 to 7 individuals in each population were selected for germination. Substrate made from coconut soil and sand (1:1 by volume) was placed into 12-cell boxes (size of one cell: 3.7 × 3.7 × 5 cm) for seed germination. One seed was sown in each cell. After sowing, the boxes were covered with a transparent top and placed in a climate room (20 °C). The sowed seeds were watered by means of a small sprayer.

**Table 1:** Sites of origin of native and invasive populations of *Senecio vulgaris*.

Range	Population code	Location	Coordinates	
Native	Barcelona	Barcelona, Spain	Lat 41.67	Long 2.73
	Pulawy	Puławy, Poland	Lat 51.40	Long 21.96
	St. Andrews	St. Andrews, UK	Lat 56.33	Long −2.78
	Fribourg	Fribourg, Switzerland	Lat 46.79	Long 7.15
	Obidos	Óbidos, Portugal	Lat 39.36	Long −9.16
	Potsdam	Potsdam, Germany	Lat 52.40	Long 13.07
Invasive	Slj.djh	Dajiuhu, Shennongjia, China	Lat 31.49	Long 109.99
	Dl.hsj	Heishijiao, Dalian, China	Lat 38.87	Long 121.56
	Lj.lsh	Lashihai, Lijiang, China	Lat 26.9	Long 100.14
	Slj.myz	Muyuzhen, Shennongjia, China	Lat 31.46	Long 110.40
	Lj.xyl	Xianyulu, Lijiang, China	Lat 26.87	Long 100.24
	Dali.sts	Santasi, Dali, China	Lat 26.70	Long 100.15

For plant rearing, we prepared substrate as described above and added slow release fertilizer (N:P:K = 14:13:13, Osmocote, The Scotts Company, USA) along with a potting medium comprising 20 g of fertilizer and 3 kg of substrate. Once 2–4 true leaves had appeared, the plants were transplanted into bigger pots (size: 8 × 8 × 9 cm) containing the substrate and fertilizer and left to grow in a greenhouse.

When some of the plants began to flower, their first capitula were pruned. A week later, when the majority of plants had developed 5–10 capitula, they were then harvested. The shoots and roots were separated at their root crowns using secateurs. The shoots were rinsed using tap water. The fresh weight of the roots and shoots was separately measured. The samples were kept separately in plastic bags and then placed in liquid nitrogen prior to storage in a freezer at −80 °C. Following this, the samples were freeze-dried in an ALPHA 1-2 LD laboratory freeze-dryer (Martin Christ, Lower Saxony, Germany). The dry weight of the roots and shoots was measured before they were ground into a fine powder and homogenized using a vortex machine. Approximately 10 mg of the powder was placed into 2 mL Eppendorf tubes and stored at −20 °C until PA extraction.

### PA extraction and analysis

The extraction and analysis of PAs was performed as described in detail in our previous work (Joosten et al., 2010; Cheng et al., 2011). In brief, approximately 10 mg of the fine powdered plant material was used to extract PAs with 1 mL 2% formic acid solution in water. At a concentration of 1 µg mL^−1^, heliotrine was added as internal standard to the extraction solvent. The plant extract solution was shaken for 0.5 h. Solid plant material was removed by centrifugation at 2,600 rpm for 10 min and filtered through a 0.2 µm nylon membrane (Acrodisc 13 mm syringe filter; Pall Corporation, NY, USA). An aliquot of the filtered solution (25 µL) was diluted with water (975 µL) and 5 µL was injected into the LC-MS/MS system (Acquity UPLC coupled to a Quattro Premier XE tandem mass spectrometer (Waters, Milford, MA, USA)), using an Acquity BEH C18, 150 × 2.1 mm, 1.7 µm (Waters, USA) UHLPC column, maintained at 50 °C, for separation of the PAs. As mobile phase A 6.5 mM ammonia in water was used and as mobile phase B acetonitrile. An analytical run was applied, starting at 100% A which was linearly changed to 50% B in 12 min, where after the mobile phase was returned to 100% A in 0.2 min. Total run time was set at 15 min and the flow was kept at 0.4 mL min^−1^. Quantification of the extracts was performed against a calibration range of PA standards (0–500 ng mL^−1^) in a blank plant extract. Ten analytical standards were available for quantification (Table 2). The concentrations of the remaining PAs were determined semi-quantitatively by comparison of their peak area with that of a related standard as indicated in Table 2. The limit of detection (LOD) for individual PAs in leaf tissue was approximately 0.5 µg g^−1^ dry weight. LC-MS/MS analytical settings used for detection and quantification of PAs are listed in Table 2.

**Table 2:** LC-MS/MS analytical settings used for detection and quantification of pyrrolizidine alkaloids (PAs).

No.	Pyrrolizidine alkaloid	Code	Retention time (min)	Precursor mass (m/z)	Fragment mass	Collision energy	Standard available^a^	PA used for (semi) quantification
1	Senecionine	Sn	9.54	336.2	94.0; 120.0	40; 30	Y	Senecionine
2	Senecionine N-oxide	Sn.ox	6.68	352.2	94.0; 120.0	40; 30	Y	Senecionine N-oxide
3	Integerrimine	Ir	9.35	336.2	94.0; 120.0	40; 30	Y	Integerrimine
4	Integerrimine N-oxide	Ir.ox	6.55	352.2	94.0; 120.0	40; 30	Y	Integerrimine N-oxide
5	Senecivernine	Sv	9.79	336.2	94.0; 120.0	40; 30	N	Integerrimine
6	Senecivernine N-oxide	Sv.ox	6.75	352.2	94.0; 120.0	40; 30	N	Integerrimine N-oxide
7	Retrorsine	Rt	8.19	352.2	94.0; 120.0	40; 30	Y	Retrorsine
8	Retrorsine N-oxide	Rt.ox	5.74	368.2	94.0; 120.0	40; 30	Y	Retrorsine N-oxide
9	Usaramine	Us	7.98	352.2	94.0; 120.0	40; 30	N	Retrorsine
10	Usaramine N-oxide	Us.ox	5.62	368.2	94.0; 120.0	40; 30	N	Retrorsine N-oxide
11	Seneciphylline	Sp	8.76	334.2	94.0; 120.0	40; 30	Y	Seneciphylline
12	Seneciphylline N-oxide	Sp.ox	6.07	350.2	94.0; 138.0	40; 30	Y	Seneciphylline N-oxide
13	Spartioidine	St	8.58	334.2	120.0; 138.0	30; 30	N	Seneciphylline
14	Spartioidine N-oxide	St.ox	6.01	350.2	94.0; 138.0	40; 30	N	Seneciphylline N-oxide
15	Riddelliine	Rd	7.58	350.2	94.0; 138.0	40; 30	Y	Riddelliine
16	Riddelliine N-oxide	Rd.ox	5.20	366.2	94.0; 118.0	40; 30	Y	Riddelliine N-oxide
17	Unknown N-oxide 1	Unk1	4.78	366.2	94.0; 118.0	40; 30	N	Riddelliine N-oxide
18	Unknown N-oxide 2	Unk2	4.84	366.2	94.0; 118.0	40; 30	N	Riddelliine N-oxide
19	Unknown N-oxide 3	Unk3	4.88	368.2	94.0; 138.0	40; 30	N	Retrorsine N-oxide
20	Unknown N-oxide 4	Unk4	5.55	368.2	94.0; 138.0	40; 30	N	Retrorsine N-oxide
21	Unknown N-oxide 5	Unk5	5.78	368.2	94.0; 138.0	40; 30	N	Retrorsine N-oxide
22	Unknown N-oxide 6	Unk6	6.22	370.2	94.0; 138.0	40; 30	N	Retrorsine N-oxide
23	Unknown N-oxide 7	Unk7	6.57	402.2	94.0; 138.0	40; 30	N	Retrorsine N-oxide
24	Unknown N-oxide 8	Unk8	6.82	402.2	94.0; 138.0	40; 30	N	Retrorsine N-oxide

**Notes.**

a Y, standard available; N, standard not available.

### Data analysis

The Shannon index of PA diversity (*H*′) in each sample was calculated according to the formula: *H*′ =  − Σ*p*_*i*_∗ln*p*_*i*_, where *p*_*i*_ is the relative abundance of each of the 20 individual PAs in a sample. The homogeneity of PA distribution in each sample (evenness, *J*′) was calculated as: *J*′ = *H*′/ln(s), where s is the total number of occurring PAs in a sample. The calculation was conducted using the R package “vegan” (Simpson et al., 2009).

Variation in PA composition was evaluated using the concentrations of all of the 20 individual PAs detected in the shoots and roots (except usaramine N-oxide and riddelliine which were only rarely detected, see Table 3). Differences in PA composition among the populations and between the shoots and roots were evaluated using an Adonis test, a nonparametric MANOVA, in which populations and plant parts (shoots or roots) were defined as factor variables.

**Table 3:** Pyrrolizidine alkaloids (PAs) variation in roots and shoots of *Senecio vulgaris* plants from native and invasive populations and grown under greenhouse conditions.

No	Pyrrolizidine alkaloid	Code	PAs in roots				PAs in shoots				Between roots and shoots	
			Presence (%)^a^	Mean conc.^b^	Min conc.	Max conc.	Presence (%)	Mean conc.	Min conc.	Max conc.	Difference^c^ (*df* = 1, 58)	Correlation^d^ (*df* = 1, 58)
1	Senecionine	Sn	100.0	129.1	2.8	397.6	100.0	30.9	1.2	84.7	^***^	0.65^***^
2	Senecionine N-oxide	Sn.ox	100.0	1049.0	5.7	2675.2	100.0	293.9	2.9	1231.7	^***^	0.57^***^
3	Integerrimine	Ir	100.0	22.6	0.7	65.9	100.0	5.0	0.1	16.6	^***^	0.68^***^
4	Integerrimine N-oxide	Ir.ox	100.0	248.1	1.7	998.6	98.3	59.2	<LOD	242.2	^***^	0.63^***^
5	Senecivernine	Sv	30.5	1.7	<LOD	18.0	18.6	0.4	<LOD	3.0	^**^	0.68^***^
6	Senecivernine N-oxide	Sv.ox	<LOD^e^									
7	Retrorsine	Rt	94.9	2.5	<LOD	35.9	88.1	2.9	<LOD	63.2	ns	0.72^***^
8	Retrorsine N-oxide	Rt.ox	96.6	20.6	<LOD	208.8	94.9	31.6	<LOD	582.4	ns	0.45^**^
9	Usaramine	Us	<LOD									
10	Usaramine N-oxide	Us.ox	1.7	0.1	<LOD	3.4	1.7	0.2	<LOD	12.6		
11	Seneciphylline	Sp	100.0	11.5	0.4	63.6	100.0	17.1	0.3	83.5	ns	0.62^***^
12	Seneciphylline N-oxide	Sp.ox	100.0	92.3	0.9	376.1	100.0	161.5	1.3	1020.1	ns	0.50^***^
13	Spartioidine	St	93.2	1.8	<LOD	6.3	89.8	2.9	<LOD	17.5	^**^	0.66^***^
14	Spartioidine N-oxide	St.ox	98.3	17.3	<LOD	57.0	100.0	29.8	0.4	212.0	ns	0.64^***^
15	Riddelliine	Rd	5.1	0.1	<LOD	3.4	1.7	0.1	<LOD	5.2		
16	Riddelliine N-oxide	Rd.ox	45.8	0.9	<LOD	14.4	57.6	1.8	<LOD	46.1	^*^	0.48^***^
17	Unknown N-oxide 1	Unk1	32.2	0.3	<LOD	2.6	35.6	1.0	<LOD	13.5	ns	0.29 ns
18	Unknown N-oxide 2	Unk2	61.0	1.0	<LOD	7.8	74.6	3.5	<LOD	32.1	^**^	0.28 ns
19	Unknown N-oxide 3	Unk3	96.6	9.3	<LOD	20.7	76.3	1.6	<LOD	6.7	^***^	0.48^***^
20	Unknown N-oxide 4	Unk4	98.3	8.5	<LOD	27.5	100.0	30.6	0.7	148.2	^***^	0.27 ns
21	Unknown N-oxide 5	Unk5	94.9	18.7	<LOD	114.3	98.3	69.2	<LOD	259.1	^***^	0.14 ns
22	Unknown N-oxide 6	Unk6	88.1	4.5	<LOD	11.2	84.8	3.2	<LOD	19.8	^**^	0.36^*^
23	Unknown N-oxide 7	Unk7	44.1	0.6	<LOD	5.6	81.4	4.4	<LOD	33.1	^***^	0.58^***^
24	Unknown N-oxide 8	Unk8	74.6	1.5	<LOD	9.1	86.4	8.3	<LOD	37.1	^***^	0.53^***^
	Total PA			1641.8	18.4	4180.6		758.8	16.3	2781.3	^***^	0.58^***^

**Notes.**

a Presence percentage = number of root/shoot samples from which a certain individual PA was detected/number of total root/shoot sample × 100 (%).

b Unit of concentration: µg/g dry weight. For the PA N-oxides with unknown identity (entries 17–24) the concentrations are estimates, based on comparison of the peak area with that of riddellliine N-oxide (entries 17 and 18) or retrorsine N-oxide (entries 19–24).

c Difference of concentration of total PA and the individual PAs between roots and shoots was investigated by paired Wilcoxon rank tests and *P*-values of the tests are shown.

d Correlation between roots and shoots in relation to concentration of total PA and the individual PA was investigated by Spearman rank correlation tests; *R* and *P*-values of the tests are shown.

e <LOD: all samples below the limit of detection (0.1 µg/g dry weight).

Level of significance:

* *p* < 0.05

** *p* < 0.01

*** *p* < 0.001.

We visualized the variation in PA composition using a nonmetric multidimensional scaling (NMDS) method, which is analogous to a principal component analysis (PCA) or multidimensional scaling (MDS), but without distribution assumptions (Goslee & Urban, 2007). Heatmaps were constructed to show difference between populations by using the R package “pheatmap” (Kolde, 2015).

We calculated the Sn/Sp ratio from the concentration of 4 PAs using the formula: (senecionine + senecionine N-oxide)/(seneciphylline + seneciphylline N-oxide). The ratios were square root transformed and used in a Kruskal-Wallis test to assess whether the ratios differed between populations. Between-population homoscedasticity was checked using Breusch-Pagan tests.

Total PA concentration and the individual concentrations of 20 PAs was log10 transformed and then used in analysis of PA concentration. Paired Wilcoxon rank tests were used to confirm whether the concentration of total PA and the individual PAs differed between the roots and shoots, while Spearman’s rank correlation tests were conducted to investigate the correlation between roots and shoots. Breusch-Pagan tests were used to assess equality of variance between the groups. *P*-values of the results were adjusted using sequential Bonferroni method when multiple tests were performed.

To confirm whether for roots and shoots the concentration, relative abundance of individual PAs, and total PA concentration differed among populations and between ranges, nested ANOVA tests were conducted in SPSS (IBM SPSS Statistics for Windows, Version 22.0. IBM Corp., Armonk, NY, USA). Equality of variance between the groups was assessed using Levene’s tests. To conduct nested ANOVA tests, we selected the 13 PAs that had an average relative abundance of more than 1%. Concentration of PAs was log transformed. Relative abundance of PAs was calculated as individual PA percentage of the total PA concentration and root square transformed.

Except nested ANOVA tests, all analyses were performed with R version 3.1.2 (R Core Team, 2015).

## Results

### PA diversity

**Figure 1:**
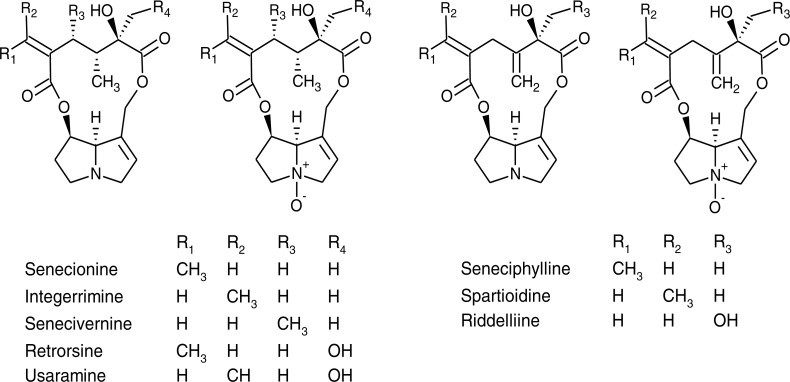
Chemical structures of pyrrolizidine alkaloids and their corresponding N-oxides identified in *Senecio vulgaris* plants.

Of the 21 PAs reported from *S. vulgaris* in the literature, 16 PAs were included in the mass spectrometric method and detected in our samples (Fig. 1). An additional eight putative PA N-oxides, with unknown identity were detected, of which it could be ascertained, based on their protonated molecular mass, fragmentation spectra and retention times, that they were different from the 21 PAs reported previously (Table S1). In many cases, both forms of PAs (tertiary amine and N-oxide) were detected in at least part of the samples. Exceptions were senecivernine and usaramine N-oxide that were detected in a number of samples, but their counterparts senecivernine N-oxide and usaramine were below the limit of detection in all samples. Similarly, no tertiary amine counterparts of the 8 unknown PA N-oxides could be identified. Thus, in total 22 PAs were detected (Table 3).

Senecionine, integerrimine, seneciphylline, and their respective N-oxides were present in the roots and shoots of all plants and all populations. Spartioidine, retrorsine and their respective N-oxides were found in all populations and in more than 90% of the individual root and shoot samples. Riddelliine N-oxide was detected in ten populations (83%), while senecivernine was detected in five populations (42%). Two PAs, riddelliine and usaramine N-oxide, were rarely found; usaramine N-oxide was only detected in the root and shoot extracts of one plant from a native population located in Potsdam, Germany, while riddelliine was found in two plants originating from Potsdam and in one plant from an invasive population in Shennongjia, China. Six of the eight unidentified PA N-oxides were found in all populations, and three of them (unk 3-5) were found in more than 90% of the shoot and root samples (Table 3, Tables S2–S3).

### Variation in PA composition

**Figure 2:**
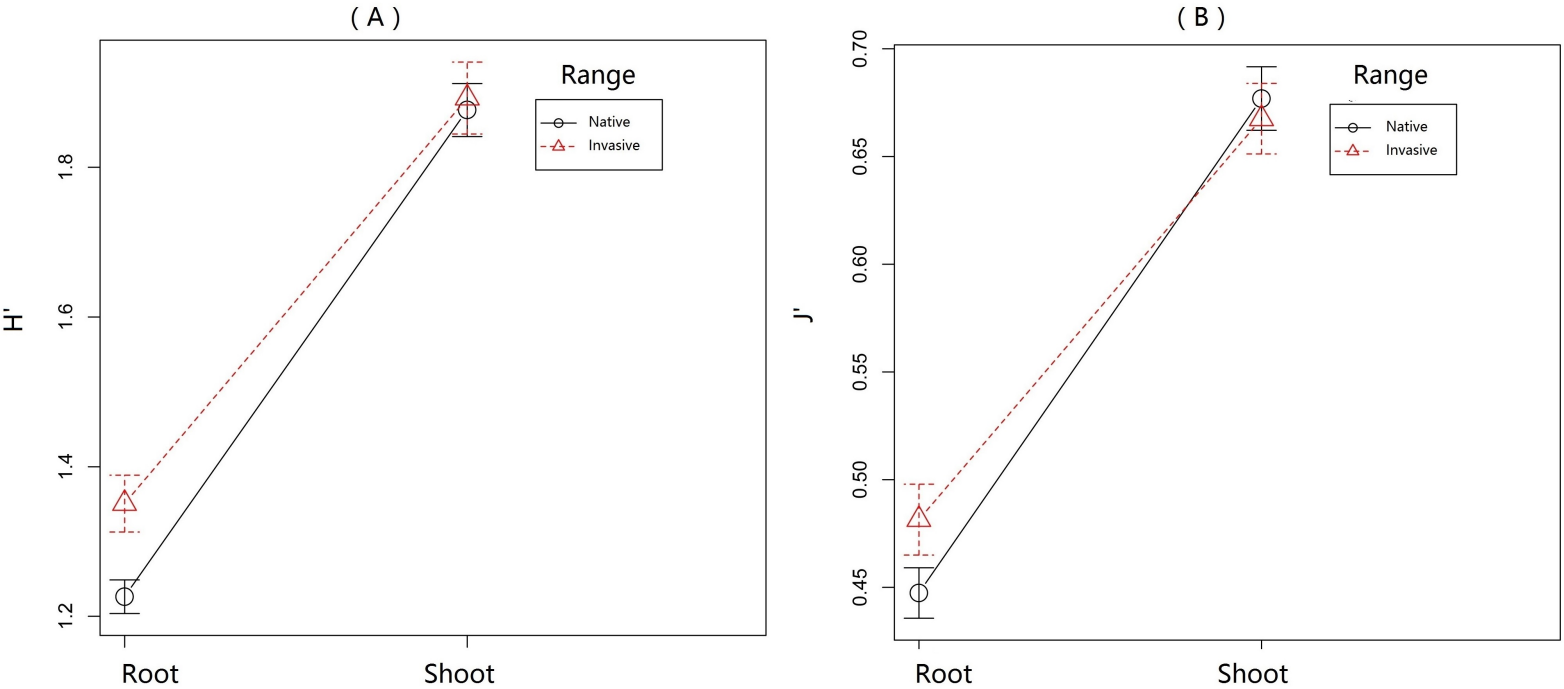
Variation of pyrrolizidine alkaloids (PAs) in roots and shoots of *Senecio vulgaris* from native and invasive populations. PA diversity was calculated as Shannon index [*H*′ =  − Σ*pi*∗ln*pi*], where *p* was the relative abundance of each of the 22 individual PAs in a sample. Homogeneity of PA distribution in each sample was calculated as evenness [*J*′ = *H*′∕ln(s)], where s was the total number of occurring PAs in a sample.

**Figure 3:**
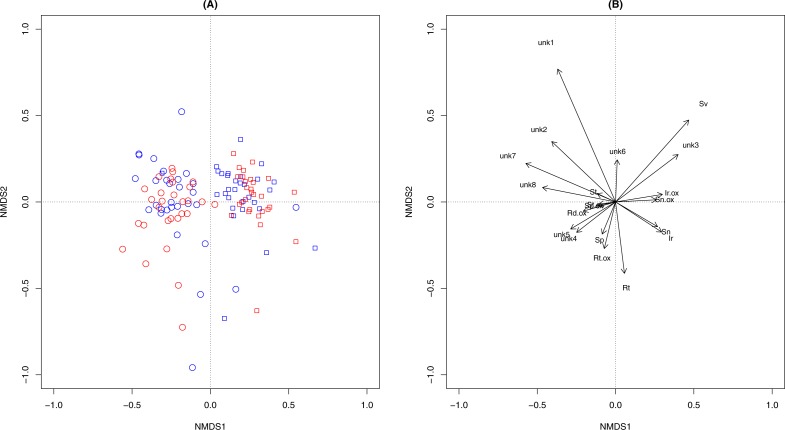
Variation of pyrrolizidine alkaloids (PAs) in roots and shoots of *Senecio vulgaris* from native and invasive populations. (A) Scoring plotting by two-dimension nonparametric multidimensional scaling (NMDS) based on concentration of 20 individual PAs. Square, roots; Dots, shoots. Red symbols were plants from invasive and the blue symbols were from native populations.(B) Loading plots of the NMDS. See details of the abbreviation of PAs in Tables 2–3.

**Figure 4:**
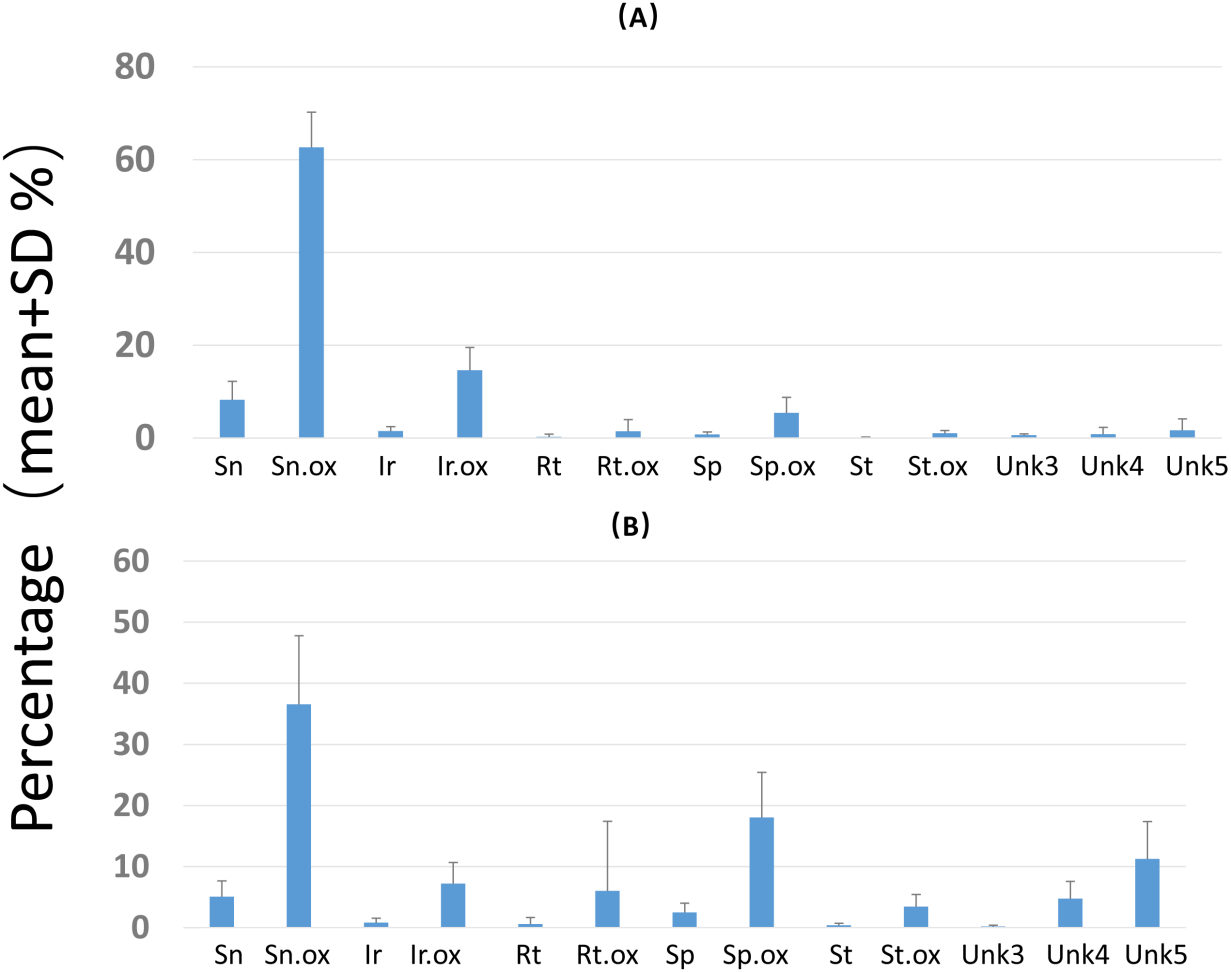
Composition of pyrrolizidine alkaloids (PAs) in roots and shoots of *Senecio vulgaris* plants. Percentage = concentration of an individual PA/total PA concentration × 100. See details of the PAs in Tables 2–3.

**Figure 5:**
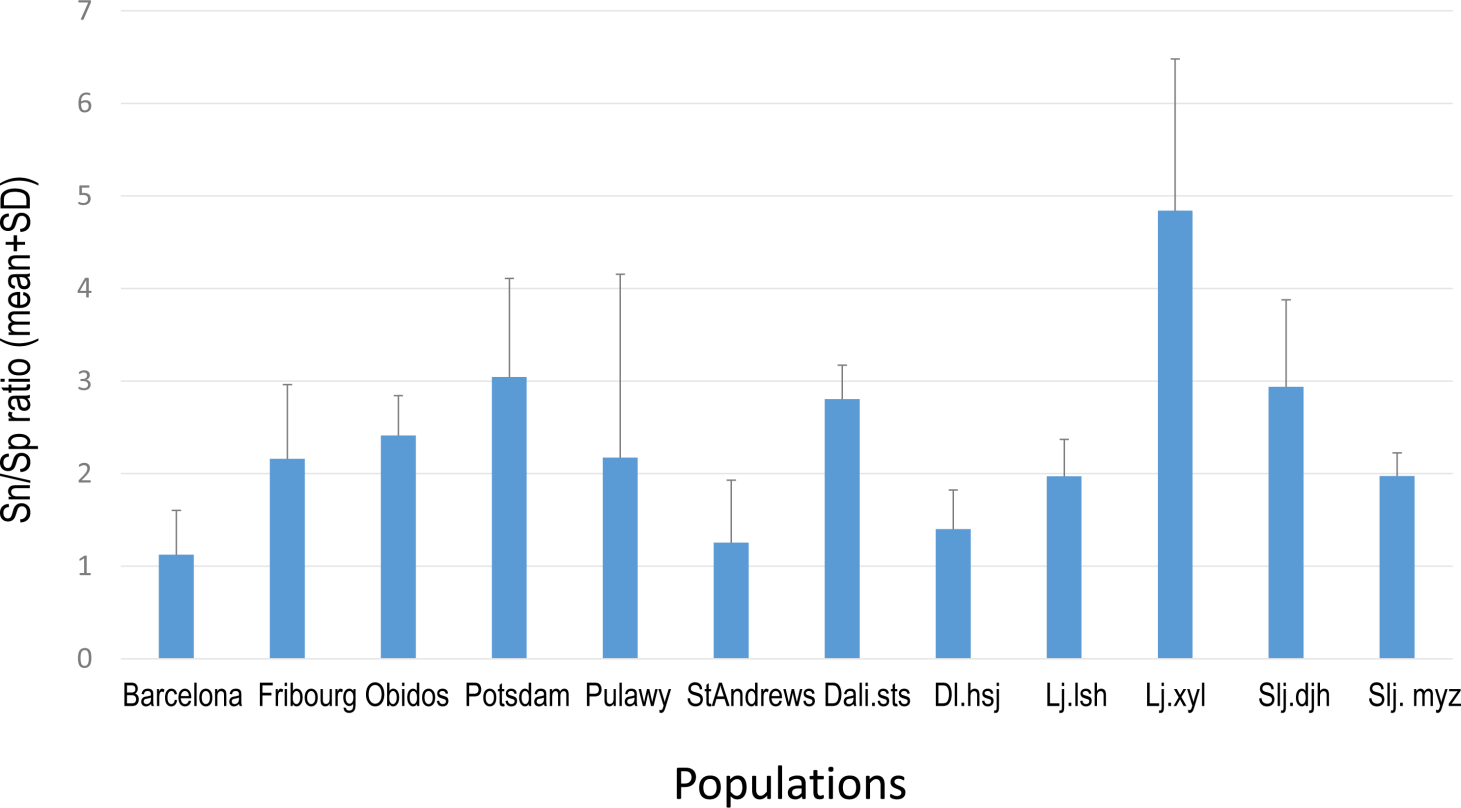
Sn/Sp ratio in shoots of *Senecio vulgaris.* plants from native and invasive populations Sn/Sp ratio = (Senecionine + Senicionine N-oxide)/(Seneciphylline + Seneciphylline N-oxide). See details of the populations in Table 1.

Overall PA diversity (*H*′) as well as evenness (*J*′) was higher in shoots than in roots, and lower in the invasive populations than in native ones (Fig. 2). Differences in PA composition were significant between organs (shoots and roots) and among populations (two factor Adonis test; organ: *df* = 1, *r*^2^ = 0.41, *p* = 0.005; populations: *df* = 11, *r*^2^ = 0.19, *p* = 0.005; Fig. 3). Senecionine N-oxide was the dominant component of the PA profile in the roots, followed by integerrimine N-oxide, senecionine and seneciphylline N-oxide (Fig. 4A). In the shoots, the four above mentioned PAs were also prevalent, in combination with retrorsine N-oxide and two unidentified PA N-oxides (unk 4 and 5, Fig. 4B). The ratio between the concentration of senecionine and that of seneciphylline (Sn/Sp ratio, including the free base and N-oxide forms of these PAs) for individual plants ranged from 0.56 to 6.87; the ratio at population level was greater than 1 and differed significantly between populations (ANOVA test: *df* = 11 and 43, *F* = 9.7, *P* < 0.001, Fig. 5).

**Figure 6:**
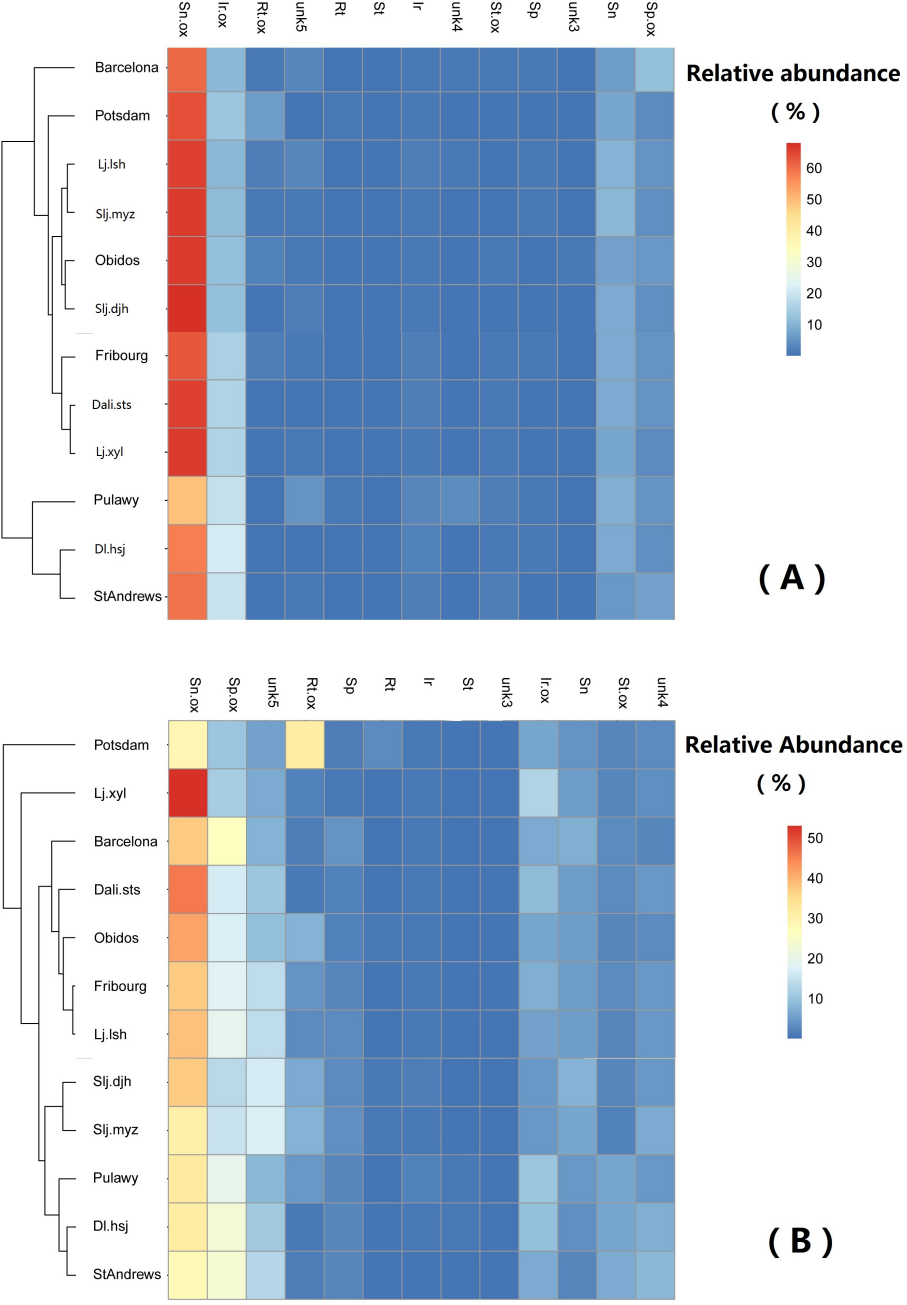
Comparison of abundance of selected pyrrolizidine alkaloids (PAs) in roots and shoots of *Senecio vulgare* plants grown under uniform conditions in the greenhouse. Plants were grew from seeds collected from 6 native and 6 invasive populations. Clustering algorithm and Euclidean distance metric were used on relative abundance values. See details of key to populations (at leaf of heatmap) and to PAs (on the top of heatmap) in Tables 1–3. The relative abundance of the 13 selected PAs was at least 1%, averaged among all samples.

Generally, the relative abundance of individual PAs was significantly different among populations (Table 4). However, the clustering of the populations did not show any geographically related pattern (Fig. 6).

**Table 4:** Results of the nested ANOVA tests of difference among *Senecio vulgaris* populations and ranges (native or invasive) for 13 selected pyrrolizidine alkaloids (PAs).

PA code	Root		Shoot	
	Population (range)	Range	Population (range)	Range
Concentration of PAs^a,b^				
Sn	1.24^ns^	0.17^ns^	1.12^ns^	0.37^ns^
Sn.ox	2.23^*^	<0.00^ns^	1.82^ns^	0.33^ns^
Ir	0.12^ns^	1.48^ns^	1.34^ns^	0.29^ns^
Ir.ox	2.23^*^	0.13^ns^	2.59^*^	0.69^ns^
Rt	2.98^**^	13.05^**^	3.26^**^	12.98^***^
Rt.ox	3.22^**^	14.15^***^	2.30^**^	14.2^***^
Sp	1.18^ns^	0.59^ns^	0.81^ns^	1.49^ns^
Sp.ox	2.23^ns^	1.26^*^	1.62^ns^	1.37^ns^
St	2.21^*^	2.49^ns^	2.30^*^	3.03^ns^
St.ox	3.14^**^	2.87^ns^	3.13^**^	3.32^ns^
Unk3	1.82^ns^	0.42^ns^	2.01^ns^	2.40^ns^
Unk4	3.12^**^	2.39^ns^	4.02^**^	0.49^ns^
Unk5	3.02^**^	1.20^ns^	2.00^ns^	0.10^ns^
Total PA concentration	2.05^*^	0.11^ns^	1.81^ns^	1.48^ns^
Relative abundance of PAs^a,c^				
Sn	0.35^ns^	1.97^ns^	2.12^*^	0.004^ns^
Sn.ox	2.68^*^	6.67^*^	2.10^*^	1.64^ns^
Ir	2.56^*^	0.16^ns^	1.46^ns^	0.51^ns^
Ir.ox	6.25^**^	0.09^ns^	2.95^**^	0.004^ns^
Rt	2.21^**^	7.96^**^	3.66^**^	2.39^ns^
Rt.ox	3.33^ns^	9.99^**^	5.44^***^	3.27^ns^
Sp	1.01^*^	0.76^ns^	1.60^ns^	0.02^ns^
Sp.ox	6.67^ns^	2.83^**^	2.27^*^	1.06^ns^
St	1.74^***^	1.25^ns^	2.83^**^	0.57^ns^
St.ox	6.34^ns^	15.51^**^	10.93^***^	5.09^ns^
Unk3	0.73^***^	0.49^ns^	0.56^ns^	0.68^ns^
Unk4	4.13^***^	1.78^ns^	2.45^*^	2.67^ns^
Unk5	3.22^**^	1.39^ns^	2.05^*^	5.48^ns^

**Notes.**

a Nested ANVOA tests were conducted separately for each individual PA (or total PA concentration) from root and shoot samples. Concentration or relative abundance of PAs were used as independent variable, population nested in ranges (*df* = 10) and range (*df* = 1) as fixed factors. In total 59 individual plants were used, and they were from 6 native and 6 invasive populations. The relative abundance of the 13 selected PAs was at least 1%, averaged among all samples.

b Concentration of PAs was calculated as µg/g dry weight and log transformed for the tests.

c Relative abundance of PAs was calculated as individual PA percentage of total PA concentration and root square transformed for the tests.

Level of significance:

ns *P* > 0.05

* *p* < 0.05

** *p* < 0.01

*** *p* < 0.001.

### Variation in PA concentration

Within the plants, significantly higher concentrations of senecionine, integerrimine (and their N-oxides), senecivernine and 2 unidentified PA N-oxides (unk 3 and 6) were present in the roots than in the shoots, but the concentrations of spartioidine, riddelliine N-oxide and 5 unidentified PA N-oxides (unk 2, 4, 5, 7 and 8) were significantly lower (Table 3). The concentrations of seneciphylline, seneciphylline N-oxide, spartioidine N-oxide, retrorsine N-oxide and an unidentified PA N-oxide (unk 1) tended to be higher in the shoots, but statistically the differences were not significant. A significant correlation between roots and shoots was found regarding the total PA concentration, as well as between most of the individual PAs, except for some unidentified PA N-oxides (Unk 1, 2, 4. 5, Table 3).

The concentration of the individual PAs and that of total PA was generally higher in plants from the native populations than in those from the invasive populations (Tables S2–S3). The difference between populations was often significant. However, significant differences between the ranges were only found for retrorsine and retrorsine N-oxide (Table 4). These two PAs were minor compounds in the PA profile of plants from both ranges (Table 3).

## Discussion

The great asset of LC-MS/MS is that it can analyse PA tertiary amine and N-oxide forms simultaneously in a single run with high sensitivity and specificity in combination with minimal sample clean-up. However, like other mass spectrometric techniques that have evolved in recent years, such as LC-QToF-MS (Skoneczny et al., 2015) and LC-Orbitrap-MS (These et al., 2013), it requires a comprehensive set of authentic analytical standards for a full quantitative result. Furthermore, although most LC-MS techniques are capable of annotating tentative PAs–based on their fragmentation spectra and or elementary composition—to establish the chemical structure of the unknowns, additional techniques, such as NMR are required.

It has been reported that PA profiles of the aboveground parts of *S. vulgaris* plants comprise seneciphylline, senecionine, retrorsine and the corresponding E-geometrical isomers, spartioidine, integerrimine and usaramine (Hartmann & Zimmer, 1986; Pieters & Vlietinck, 1988). In *S. vulgaris* PAs are primarily produced as N-oxides in the roots, which is also the dominant form of PAs in the other parts of the plant (Hartmann & Dierich, 1998). Apart from the PAs mentioned above, riddelliine, senecivernine, platyphylline and neoplatyphylline have been reported in the aerial parts of *S. vulgaris* plants (Von Borstel, Witte & Hartmann, 1989; Yang et al., 2011), as well as neosenkirkine (Von Borstel, Witte & Hartmann, 1989) and othonnine (Xiong et al., 2012). The 21 PAs with identified structures detected from *S. vulgaris* plants in previous studies have been summarized in Table S1 and structures of most of them were shown in Fig. 1.

Of these 21 PAs reported previously, 16 PAs were included in the mass spectrometric method, most of which were detected in this study. However, due to a lack of suitable reference standards, we were unable to search for platyphylline, neoplatyphylline, neosenkirkine, or othonnine in the root and shoot extracts of *S. vulgaris.* However, three unidentified PA N-oxides (Unk 3-5), with the same molecular mass as retrorsine N-oxide and that could be structural isomers of the latter were present in more than 90% of the samples. In particular, one PA N-oxide (Unk 5) comprised about 10% of the total PA concentration in shoot samples, although a reliable quantification of this compound due to lack of a standard could not be made. It would be worthwhile to elucidate the structure of these three PAs and explore whether they are dominant in the PA profiles of certain *S. vulgaris* plants.

Some studies have found either senecionine (Hartmann & Zimmer, 1986) or seneciphylline to be dominant (Lüthy, Heim & Schlatter, 1983), while others have found both PAs to be dominant in *S. vulgaris* (Von Borstel, Witte & Hartmann, 1989; Brown & Molyneux, 1996). In this study, senecionine was generally present in higher concentrations than seneciphylline.

The shoots and roots of *S. vulgaris* plants differed in that shoots showed more divergent PA profiles and that the shoots had a lower total PA content than the roots. Although there were significant differences in PA variation between the shoots and roots, these parts were positively correlated regarding the concentrations of total PAs and most of individual PAs (Table 3). This pattern could be explained by the processes of PA synthesis and accumulation in *S. vulgaris* plants, as PAs are primarily produced as senecionine N-oxide in the roots, while structural transformation mainly occurs in the shoots. Usually there is little turnover of PAs once being produced and they translocate to plant tissues mainly via the phloem (Hartmann & Dierich, 1998). Similar patterns regarding differences and correlations of PAs between the roots and shoots have been found in *J. vulgaris* (Cheng et al., 2011; Joosten et al., 2011).

Higher PA concentrations in the belowground parts compared with the aboveground parts of *S. vulgaris* plants have been found in the vegetative stage. It has been reported that when the plants have produced buds, the highest PA concentrations are found in the capitula, while the stems and leaves generally contain lower total PA concentrations compared to the roots (Hartmann & Zimmer, 1986). This consistent with our finding that the total PA concentration was lower in the shoots than in the roots when the *S. vulgaris* plants were not yet flowering.

The indexes of PA diversity and evenness were somewhat lower for plants from invasive ranges than those from the native range (Fig. 2). This indicated that invasive *S. vulgaris* populations tended to produce less diverse PA profiles than the native ones. However, this trend is much weaker than observed in some other invasive species. For instance, native *J. vulgaris* populations expressed four chemotypes (Macel, Vrieling & Klinkhamer, 2004), while in invasive *J. vulgaris* populations one chemotype dominated (Joshi & Vrieling, 2005). PA diversity in *S. pterophorus* (native to South Africa) was reduced after introduction in Europe and Australia (Castells, Mulder & Perez-Trujillo, 2014). Furthermore, invasive *Tanacetum vulgare* plants contained a smaller number of qualitative defense compounds than the native ones (Wolf et al., 2011).

We also found that invasive *S. vulgaris* populations did not produce higher concentrations of individual and of total PAs than native populations. These results did not agree with our prediction deduced from the SDH. Some studies have found that PA levels of related species significantly increased in the invaded range. For example, invasive populations of *S. inaequidens*, *S. pterophorus* and *J. vulgaris* all showed a significantly higher total PA concentration than their native conspecifics (Joshi & Vrieling, 2005; Caño et al., 2009; Castells, Mulder & Perez-Trujillo, 2014; Lin, Klinkhamer & Vrieling, 2015). Some other invasive species appear to have evolved towards decreased chemical defense levels but they may have developed other compensatory mechanisms that contribute to their invasion success. For instance, invasive genotypes of *Sapium sebiferum* evolved a reduced defense and resistance ability, but were more tolerant and outperformed the native genotypes under higher levels of herbivore attack (Zou et al., 2008).

The prerequisite of the EICA hypothesis and SDH is that invasive plants face a lower specialist herbivore pressure in invasive ranges. We could confirm that *S. vulgaris* populations in China were attacked by insect herbivores but we did not determine whether the insects were specialists or not. Although it is likely that most insects will be generalists, it is not impossible that there may be one or more specialists among them that have adapted to *S. vulgaris*, since *S. vulgaris* has a long invasive history and more than 60 congeneric species have been identified in China (Chen, 1999). Since there are significant variations between populations, a good revisiting study on the EICA hypothesis and SDH needs enough populations for a robust statistical analysis, and it is also important to describe and cluster invasive populations by analysis of their genetic structure in the different ranges; otherwise it remains difficult to determine whether the differences between native and invasive populations are the result of evolution or of pre-adaption (Pan et al., 2013; Turner, Hufbauer & Rieseberg, 2014; Siemann et al., 2016; Schrieber et al., 2016).

Taking into account the high PA levels present in *S. vulgaris* and the toxic effect that PAs exert on most herbivores, it reasonable to assume that PAs play an important role in the chemical defense of *S. vulgaris*. However, there are also other metabolites that can function as chemical defense in *S. vulgaris*. For instance, an oplopane sesquiterpene and jacaranone were identified from *S. vulgaris* (Liu, Zhang & Wang, 2010). Both compounds (or similar compounds) have a negative effect on insect feeding (Lajide, Escoubas & Mizutani, 1996; Reina et al., 2001; Xu, Zhang & Casida, 2003). It will be interesting to investigate whether the levels of other qualitative defense compounds such as oplopane sesquiterpenes and jacaranone are higher in invasive *S. vulgaris* populations than in native ones, as the SDH would predict. It may be advantageous to use a non-targeted analysis approach to explore for metabolites of potential significance, as was recently shown in the study of Skoneczny et al. (2017).

## Conclusions

As the *Senecio vulgaris* plants from native and invasive ranges were grown under identical conditions, the differences in PA concentration and PA composition between ranges and between populations might thus be explained by their genetic variation. In our study the invasive *S. vulgaris* populations had slightly less diverse PA profiles and tended to have lower concentrations of individual PAs compared to the native populations. This finding is in contrast to the predictions of the SDH. However, the current findings should also be treated with caution given the limited number of populations sampled, the lack of background information about herbivore guilds feeding on *S. vulgaris* and the limited knowledge on the genetic structure of *S. vulgaris* populations in the different ranges. Future studies should focus on sampling a larger number of populations and screening for a wider array of plant metabolites in order to address these questions.

##  Supplemental Information

10.7717/peerj.3686/supp-1 Supplemental Information 1 Concentrations of individual and groups of pyrrolizidine alkaloids (PAs, *μ*g/g dry weight) in roots and shoots of *Senecio vulgaris* plants from native and invasive populations

10.7717/peerj.3686/supp-2 Table S1 Pyrrolizidine alkaloids (PAs) in *Senecio vulgaris* plants reported in previous works and this study * 1 = reported, 0 = not reported; See details of these studies in reference list.
** ND = not determined.
**** NP = not reported.

10.7717/peerj.3686/supp-3 Table S2 Mean concentrations of pyrrolizidine alkaloids (PAs, *μ*g/g dry weight) in roots of *Senecio vulgaris* plants from native and invasive populations (*n* = 59, ±SD) * * See Table 1 for explanation of the population code.

10.7717/peerj.3686/supp-4 Table S3 Mean concentrations of pyrrolizidine alkaloids (PAs, *μ*g/g dry weight) in shoots of *Senecio vulgaris* plants from native and invasive populations (*n* = 59, ±SD) * See Table 1 for explanation of the population code.

## Additional Information and Declarations

### Data Availability

The following information was supplied regarding data availability: The raw data has been supplied as Supplemental dataset.

## References

[ref-1] Barlow VM, Godfrey LD, Norris RF (1999). Population dynamics of *Lygus hesperus* (Heteroptera: Miridae) on selected weeds in comparison with alfalfa. Journal of Economic Entomology.

[ref-2] Blossey B, Notzold R (1995). Evolution of increased competitive ability in invasive nonindigenous plants: a hypothesis. Journal of Ecology.

[ref-3] Brown MS, Molyneux RJ (1996). Effects of water and mineral nutrient deficiencies on pyrrolizidine alkaloid content of *Senecio vulgaris* flowers. Journal of the Science of Food and Agriculture.

[ref-4] Caño L, Escarre J, Vrieling K, Sans FX (2009). Palatability to a generalist herbivore, defence and growth of invasive and native Senecio species: testing the evolution of increased competitive ability hypothesis. Oecologia.

[ref-5] Castells E, Mulder PP, Perez-Trujillo M (2014). Diversity of pyrrolizidine alkaloids in native and invasive Senecio pterophorus (Asteraceae): implications for toxicity. Phytochemistry.

[ref-6] Cates RG (1980). Feeding patterns of monophagous, oligophagous, and polyphagous insect herbivores: the effect of resource abundance and plant chemistry. Oecologia.

[ref-7] Catford JA, Jansson R, Nilsson C (2009). Reducing redundancy in invasion ecology by integrating hypotheses into a single theoretical framework. Diversity and Distributions.

[ref-8] Chen Y (1999). Asteraceae: flora reipublicae popularis sinicae (in Chinese).

[ref-9] Cheng D, Kirk H, Mulder PPJ, Vrieling K, Klinkhamer PGL (2011). Pyrrolizidine alkaloid variation in shoots and roots of segregating hybrids between *Jacobaea vulgaris* and *Jacobaea aquatica*. New Phytologist.

[ref-10] Doorduin LJ, Vrieling K (2011). A review of the phytochemical support for the shifting defence hypothesis. Phytochemistry Reviews.

[ref-11] Feeny P (1976). Plant apparency and chemical defense. Recent Advances in Phytochemistry.

[ref-12] Frantzen J, Hatcher P (1997). A fresh view on the control of the annual plant *Senecio vulgaris*. Integrated Pest Management Reviews.

[ref-13] Goslee SC, Urban DL (2007). The ecodist package for dissimilarity-based analysis of ecological data. Journal of Statistical Software.

[ref-14] Handley RJ, Steinger T, Treier UA, Mueller-Schaerer H (2008). Testing the evolution of increased competitive ability (EICA) hypothesis in a novel framework. Ecology.

[ref-15] Hartmann T, Dierich B (1998). Chemical diversity and variation of pyrrolizidine alkaloids of the senecionine type: biological need or coincidence?. Planta.

[ref-16] Hartmann T, Zimmer M (1986). Organ-specific distribution and accumulation of pyrrolizidine alkaloids during the life history of two annual *Senecio* species. Journal of Plant Physiology.

[ref-17] Joosten L, Cheng D, Mulder PPJ, Vrieling K, Van Veen J, Klinkhamer PGL (2011). The genotype dependent presence of pyrrolizidine alkaloids as tertiary amine in *Jacobaea vulgaris*. Phytochemistry.

[ref-18] Joosten L, Mulder PPJ, Vrieling K, Van Veen J, Klinkhamer PGL (2010). The analysis of pyrrolizidine alkaloids in *Jacobaea vulgaris*; a comparison of extraction and detection methods. Phytochemical Analysis.

[ref-19] Joosten L, Van Veen JA (2011). Defensive properties of pyrrolizidine alkaloids against microorganisms. Phytochemistry Reviews.

[ref-20] Joshi J, Vrieling K (2005). The enemy release and EICA hypothesis revisited: incorporating the fundamental difference between specialist and generalist herbivores. Ecollogy Letters.

[ref-21] Kadereit JW (1984). The origin of *Senecio vulgaris* (Asteraceae). Plant Systematics and Evolution.

[ref-22] Keane RM, Crawley MJ (2002). Exotic plant invasions and the enemy release hypothesis. Trends in Ecology & Evolution.

[ref-23] Kleine S, Mülller C (2010). Intraspecific plant chemical diversity and its relation to herbivory. Oecologia.

[ref-24] Kolde R (2015). https://cran.r-project.org/web/packages/pheatmap/.

[ref-25] Lajide L, Escoubas P, Mizutani J (1996). Cyclohexadienones-insect growth inhibitors from the foliar surface and tissue extracts of *Senecio cannabifolius*. Cellular & Molecular Life Sciences.

[ref-26] Li Z, Xie Y (2002). Invasive alien species in China (in Chinese).

[ref-27] Lin T, Klinkhamer PGL, Vrieling K (2015). Parallel evolution in an invasive plant: effect of herbivores on competitive ability and regrowth of *Jacobaea vulgaris*. Ecology Letters.

[ref-28] Liu Y, Zhang Z, Wang Y (2010). Chemical constituents in *Senecio vulgaris* (in Chinese with English abstract). Chinese Traditional and Herbal Drugs.

[ref-29] Lüthy J, Heim T, Schlatter C (1983). Transfer of [3H] pyrrolizidine alkaloids from *Senecio vulgaris* L. and metabolites into rat milk and tissues. Toxicology Letters.

[ref-30] Macel M, Bruinsma M, Dijkstra SM, Ooijendijk T, Niemeyer HM, Klinkhamer PGL (2005). Differences in effects of pyrrolizidine alkaloids on five generalist insect herbivore species. Journal of Chemical Ecology.

[ref-31] Macel M, Vrieling K, Klinkhamer PGL (2004). Variation in pyrrolizidine alkaloid patterns of *Senecio jacobaea*. Phytochemistry.

[ref-32] Minkenberg O, Lenteren JC (1986). The leafminers, *Liriomyza bryoniae* and *L. trifolii* (Diptera: Agromyzidae), their parasites and host plants: a review. Agricultural University Wageningen Papers.

[ref-33] Müller-Scharer H, Schaffner U, Steinger T (2004). Evolution in invasive plants: implications for biological control. Trends in Ecology & Evolution.

[ref-34] Pan XY, Jia X, Fu DJ, Li B (2013). Geographical diversification of growth-defense strategies in an invasive plant. Journal of Systematics and Evolution.

[ref-35] Pieters LA, Vlietinck AJ (1988). Spartioidine and usaramine, two pyrrolizidine alkaloids from *Senecio vulgaris*. Planta Medica.

[ref-36] R Core Team. (2015).

[ref-37] Reina M, González-coloma A, Gutiérrez C, Cabrera R, Rodríguez ML, Fajardo V, Luis V (2001). Defensive chemistry of *Senecio miser*. Journal of Natural Products.

[ref-38] Rhoades DF, Cates RG (1976). Toward a general theory of plant antiherbivore chemistry. Recent Advances in Phytochemistry.

[ref-39] Robinson DE, O’Donovan JT, Sharma MP, Doohan DJ, Figueroa R (2003). The biology of Canadian weeds. 123. *Senecio vulgaris* L. Canadian Journal of Plant Science.

[ref-40] Schrieber K, Wolf S, Wypior C, Höhlig D, Hensen I, Lachmuth S (2016). Adaptive and non-adaptive evolution of trait means and genetic trait correlations for herbivory resistance and performance in an invasive plant. Oikos.

[ref-41] Siemann E, Dewalt SJ, Zou J, Rogers WE (2016). An experimental test of the EICA hypothesis in multiple ranges: invasive populations outperform those from the native range independent of insect herbivore suppression. AoB Plants.

[ref-42] Simpson GL, Solymos P, Stevens M, Wagner H (2009). Vegan: community ecology package. Time International.

[ref-43] Skoneczny D, Weston PA, Zhu X, Gurr GM, Callaway RM, Barrow RA, Weston LA (2017). Metabolic profiling and identification of shikonins in root periderm of two invasive *Echium* spp. weeds in Australia. Molecules.

[ref-44] Skoneczny D, Weston PA, Zhu XC, Gurr GM, Callaway RM, Weston LA (2015). Metabolic profiling of pyrrolizidine alkaloids in foliage of two *Echium* spp. invaders in Australia–a case of novel weapons?. International Journal of Molecular Sciences.

[ref-45] Stegelmeier BL, Edgar JA, Colegate SM, Gardner DR, Schoch TK, Coulombe RA, Molyneux RJ (1999). Pyrrolizidine alkaloid plants, metabolism and toxicity. Journal of Natural Toxins.

[ref-46] These A, Bodi D, Ronczka S, Lahrssen-Wiederholt M, Preiss-Weigert A (2013). Structural screening by multiple reaction monitoring as a new approach for tandem mass spectrometry: presented for the determination of pyrrolizidine alkaloids in plants. Analytical Bioanalytical Chemistry.

[ref-47] Trigo JR (2011). Effects of pyrrolizidine alkaloids through different trophic levels. Phytochemistry Reviews.

[ref-48] Turner KG, Hufbauer RA, Rieseberg LH (2014). Rapid evolution of an invasive weed. New Phytologist.

[ref-49] Van Dam NM, Vuister LWM, Bergshoeff C, De Vos H, Vander Meijden ED (1995). The ‘raison d’etre’ of pyrrolizidine alkaloids in *Cynoglossum officinale* deterrent effects against generalist herbivores. Journal of Chemical Ecology.

[ref-50] Van der Meijden E (1996). Plant defence, an evolutionary dilemma: contrasting effects of (specialist and generalist) herbivores and natural enemies. Entomologia Experimentalis et Applicata.

[ref-51] Vilà M, Hulme PE (2017). Impact of biological invasions on ecosystem services.

[ref-52] Von Borstel K, Witte L, Hartmann T (1989). Pyrrolizidine alkaloid patterns in populations of *Senecio vulgaris*, *Senecio vernalis* and their hybrids. Phytochemistry.

[ref-53] Wolf VC, Berger U, Gassmann A, Müller C (2011). High chemical diversity of a plant species is accompanied by increased chemical defence in invasive populations. Biological Invasions.

[ref-54] Xiong A, Yang L, Ji L, Wang Z, Yang X, Chen Y, Wang X, Wang C, Wang Z (2012). UPLC-MS based metabolomics study on *Senecio scandens* and *S. vulgaris*: an approach for the differentiation of two *Senecio* herbs with similar morphology but different toxicity. Metabolomics.

[ref-55] Xu H, Qiang S, Genovesi P, Ding H, Wu J, Meng L, Han Z, Miao J, Hu B, Guo J (2012). An inventory of invasive alien species in China. NeoBiota.

[ref-56] Xu H, Zhang N, Casida JE (2003). Insecticides in Chinese medicinal plants: survey leading to jacaranone, a neurotoxicant and glutathione-reactive quinol. Journal of Agricultural and Food Chemistry.

[ref-57] Yang X, Yang L, Xiong A, Li D, Wang Z (2011). Authentication of Senecio scandens and *S. vulgaris* based on the comprehensive secondary metabolic patterns gained by UPLC-DAD/ESI-MS. Journal of Pharmaceutical and Biomedical Analysis.

[ref-58] Zhu BR, Barrett SCH, Zhang DY, Liao WJ (2017). Invasion genetics of *senecio vulgaris*: loss of genetic diversity characterizes the invasion of a selfing annual, despite multiple introductions. Biological Invasions.

[ref-59] Zou J, Siemann E, Rogers WE, DeWalt SJ (2008). Decreased resistance and increased tolerance to native herbivores of the invasive plant *Sapium sebiferum*. Ecography.

