# Supplementary material for: Pyrrolizidine alkaloid variation in Senecio vulgaris populations from native and invasive ranges
Source: PeerJ. 2017 Aug 14;5:e3686. doi: 10.7717/peerj.3686 (PMC5560238; doi:10.7717/peerj.3686)
Supplement: Table S1 — * 1 = reported, 0 = not reported; See details of these studies in reference list. ** ND = not determined. **** NP = not reported. [file peerj-05-3686-s002.docx]

| No. | Pyrrolizidine alkaloid | RT (min) | MH+ (m/z) | Previous Studies * | | | | This study | Times reported |
| --- | --- | --- | --- | --- | --- | --- | --- | --- | --- |
|  |  |  |  | Hartmann 1986 | von Borstel 1989 | Yang  2011 | Xiong  2012 |  |  |
| 1 | senecionine | 9.54 | 336.2 | 1 | 1 | 1 | 1 | 1 | 5 |
| 2 | senecionine *N*-oxide | 6.68 | 352.2 | 1 | 1 | 1 | 1 | 1 | 5 |
| 3 | integerrimine | 9.35 | 336.2 | 1 | 1 | 1 | 0 | 1 | 4 |
| 4 | integerrimine *N*-oxide | 6.55 | 352.2 | 1 | 1 | 1 | 1 | 1 | 5 |
| 5 | retrorsine | 8.19 | 352.2 | 1 | 1 | 1 | 1 | 1 | 5 |
| 6 | retrorsine *N*-oxide | 5.74 | 368.2 | 1 | 1 | 1 | 1 | 1 | 5 |
| 7 | usaramine | 7.98 | 352.2 | 0 | 0 | 1 | 0 | 0 | 1 |
| 8 | usaramine *N*-oxide | 5.62 | 368.2 | 0 | 0 | 0 | 0 | 1 | 1 |
| 9 | riddelliine | 7.58 | 350.2 | 0 | 1 | 1 | 0 | 1 | 3 |
| 10 | riddelliine *N*-oxide | 5.20 | 366.2 | 0 | 1 | 0 | 0 | 1 | 2 |
| 11 | seneciphylline | 8.76 | 334.2 | 1 | 1 | 1 | 1 | 1 | 5 |
| 12 | seneciphylline *N*-oxide | 6.07 | 350.2 | 1 | 1 | 1 | 1 | 1 | 5 |
| 13 | spartioidine | 8.58 | 334.2 | 0 | 1 | 1 | 0 | 1 | 3 |
| 14 | spartioidine *N*-oxide | 6.01 | 350.2 | 0 | 1 | 1 | 0 | 1 | 3 |
| 15 | senecivernine | 9.79 | 336.2 | 0 | 1 | 0 | 0 | 1 | 2 |
| 16 | unknown N-oxide 1 | 4.78 | 366.2 | 0 | 0 | 0 | 0 | 1 | 1 |
| 17 | unknown N-oxide 2 | 4.84 | 366.2 | 0 | 0 | 0 | 0 | 1 | 1 |
| 18 | unknown N-oxide 3 | 4.88 | 368.2 | 0 | 0 | 0 | 0 | 1 | 1 |
| 19 | unknown N-oxide 4 | 5.55 | 368.2 | 0 | 0 | 0 | 0 | 1 | 1 |
| 20 | unknown N-oxide 5 | 5.78 | 368.2 | 0 | 0 | 0 | 0 | 1 | 1 |
| 21 | unknown N-oxide 6 | 6.22 | 370.2 | 0 | 0 | 0 | 0 | 1 | 1 |
| 22 | unknown N-oxide 7 | 6.57 | 402.2 | 0 | 0 | 0 | 0 | 1 | 1 |
| 23 | unknown N-oxide 8 | 6.82 | 402.2 | 0 | 0 | 0 | 0 | 1 | 1 |
| 24 | neosenkirkine | ND** | 366.2 | 0 | 0 | 1 | 0 | 0 | 1 |
| 25 | neoplatyphylline | ND | 338.2 | 0 | 1 | 1 | 0 | 0 | 2 |
| 26 | platyphylline | ND | 338.2 | 0 | 1 | 1 | 1 | 0 | 3 |
| 27 | othonnine | ND | 354.2 | 0 | 0 | 0 | 1 | 0 | 1 |
| 28 | unknown 9 | ND | 334.2 | 0 | 1 | 0 | 0 | 0 | 1 |
| 29 | unknown10 | ND | 336.2 | 0 | 0 | 1 | 0 | 0 |  |
| total number of PAs | |  |  | 8 | 15 | 15 | 9 | 22 | 29 |
| number of populations used | |  |  | NP*** | 10 | NP | 6 | 12 | 28 |
