# Supplementary material for: Pyrrolizidine alkaloid variation in Senecio vulgaris populations from native and invasive ranges
Source: PeerJ. 2017 Aug 14;5:e3686. doi: 10.7717/peerj.3686 (PMC5560238; doi:10.7717/peerj.3686)
Supplement: Table S2 — * See Table 1 for explanation of the population code. [file peerj-05-3686-s003.docx]

| No. | Pyrrolizidine alkaloid | Native populations * | | | | | | Invasive populations * | | | | | |
| --- | --- | --- | --- | --- | --- | --- | --- | --- | --- | --- | --- | --- | --- |
|  |  | Barcelona  （n=5 ) | Fribourg ( n=5 ) | Óbidos ( n=5) | Potsdam ( n=5) | Puławy ( n=5 ) | StAndrews ( n=5) | Dali.sts ( n=5 ) | Dl.hsj ( n=5 ) | Lj.lsh ( n=5 ) | Lj.xyl ( n=4 ) | Slj.djh ( n=5 ) | Slj.myz ( n=5) |
| 1 | senecionine | 137±151 | 148±90 | 133±98 | 126±89 | 41.3±32.8 | 153±68 | 177±133 | 113±72 | 100±78 | 197±126 | 116±64 | 124±99 |
| 2 | senecionine N-oxide | 1123 ±717 | 1199±508 | 1126±491 | 1007±617 | 299±265 | 1574±644 | 1336±668 | 990±643 | 684±523 | 1570±468 | 923±235 | 863±836 |
| 3 | integerrimine | 18±19 | 27±12 | 18±14 | 20±16 | 10.8±7.6 | 40±19 | 30±22 | 31±18 | 13.4±10.5 | 34±23 | 16.2±8.9 | 16±13 |
| 4 | integerrimine N-oxide | 196±138 | 302±181 | 205±93 | 205±150 | 97±66 | 522±280 | 328±170 | 363±225 | 110±84 | 360±55 | 161±33 | 150±151 |
| 5 | senecivernine | 0.6±1.4 | <LOD | 4.5±3.8 | <LOD | <LOD | 7.6±6.8 | <LOD | <LOD | <LOD | <LOD | 3.8±1.7 | 3.8±4.0 |
| 6 | retrorsine | 1.6±0.6 | 2.7±0.9 | 4.9±4.3 | 11.1±14.2 | 1.0±0.6 | 1.3±0.6 | 1.0±0.5 | 0.6±0.8 | 1.2±0.8 | 1.7±1.1 | 1.2±0.7 | 2.2±2.9 |
| 7 | retrorsine N-oxide | 20±21 | 26±13 | 41±22 | 91±100 | 3.9±2.9 | 11.9±3.0 | 7.9±4.7 | 4.1±3.2 | 9.6±8.3 | 11.9±7.6 | 8.6±4.2 | 10.1±7.1 |
| 8 | seneciphylline | 24±24 | 15.1±14.9 | 12.1±9.3 | 8.2±6.1 | 4.5±3.7 | 16.2±8.3 | 11.0±7.1 | 9.8±5.5 | 8.7±6.7 | 9.5±3.2 | 9.0±7.2 | 9.4±7.2 |
| 9 | seneciphylline N-oxide | 208±145 | 93±13 | 97±46 | 65±53 | 29±27 | 166±25 | 96±49 | 84±56 | 59±53 | 88±13 | 61±31 | 62±65 |
| 10 | spartioidine | 2.2±1.4 | 1.9±1.0 | 1.5±1.0 | 1.2±1.0 | 1.3±1.0 | 4.3±2.1 | 1.5±0.8 | 2.4±1.3 | 1.1±0.9 | 1.8±0.3 | 0.9±0.6 | 1.0±0.9 |
| 11 | spartioidine N-oxide | 22±12 | 19.6±8.4 | 14.2±6.2 | 9.2±5.8 | 9.2±6.8 | 47±7.6 | 19±10 | 25±15 | 6.7±5.5 | 21.3±4.5 | 8.6±3.9 | 7.4±7.1 |
| 12 | riddelliine N-oxide | 1.0±1.9 | 0.7±0.4 | 1.0±0.7 | 5.1±6.6 | 0.3±0.4 | 0.7±0.5 | <LOD | 0.1±0.2 | 0.3±0.6 | <LOD | 0.5±0.6 | 0.5±0.6 |
| 13 | unknown N-oxide 1 | 0.4±0.6 | 0.3±0.5 | <LOD | 0.1±0.3 | 0.1±0.2 | 1.0±0.7 | 0.3±0.4 | 0.3±0.5 | 0.5±1.2 | 0.1±0.2 | 0.2±0.3 | <LOD |
| 14 | unknown N-oxide 2 | 3.0±2.8 | 0.7±0.7 | 0.5±0.7 | 0.7±0.9 | 0.5±0.5 | 2.5±1.8 | 0.6±0.9 | 0.6±0.8 | 0.3±0.7 | 0.4±0.3 | 0.9±0.8 | 0.8±0.9 |
| 15 | unknown N-oxide 3 | 9.3±4.4 | 12.0±5.3 | 12.5±2.4 | 7.2±5.1 | 3.7±2.5 | 10.2±6.1 | 9.3±4.2 | 10.6±5.7 | 7.1±4.6 | 16.0±3.6 | 8.2±2.1 | 6.7±5.7 |
| 16 | unknown N-oxide 4 | 14.3±7.7 | 8.0±1.8 | 9.9±3.3 | 3.5±2.8 | 8.2±4.8 | 14.4±2.3 | 5.5±3.2 | 6.5±3.7 | 5.5±3.2 | 11.9±2.1 | 8.9±2.0 | 6.4±4.8 |
| 17 | unknown N-oxide 5 | 46±38 | 18.1±2.8 | 19.4±7.0 | 8.2±8.0 | 13.5±10.7 | 26.5±3.5 | 10.9±6.7 | 9.9±6.1 | 12.5±6.8 | 21.7±4.7 | 22.5±6.6 | 14.8±12.9 |
| 18 | unknown N-oxide 6 | 4.0±2.3 | 5.4±3.1 | 5.9±2.0 | 2.7±2.3 | 4.2±3.2 | 8.7±2.2 | 3.2±2.4 | 3.5±2.0 | 2.0±1.4 | 6.0±1.1 | 4.8±1.5 | 3.6±3.5 |
| 19 | unknown N-oxide 7 | 0.7±0.7 | 0.5±0.7 | 0.3±0.5 | 0.1±0.3 | 0.4±0.6 | 3.0±1.8 | 0.5±0.5 | 1.0±0.9 | <LOD | 0.3±0.4 | 0.1±0.2 | 0.3±0.4 |
| 20 | unknown N-oxide 8 | 4.2±3.2 | 1.7±1.4 | 1.3±0.8 | 0.6±0.6 | 0.8±0.9 | 3.0±2.3 | 0.8±0.6 | 1.6±1.5 | 0.6±0.7 | 0.3±0.5 | 1.3±0.4 | 1.2±1.2 |
| Total PA concentration | | 1835±1117 | 1880±675 | 1707±741 | 1574±905 | 530±424 | 2613±944 | 2038±1024 | 1656±1026 | 1022±750 | 2351±659 | 1357±305 | 1283±1195 |
