# Supplementary material for: Pyrrolizidine alkaloid variation in Senecio vulgaris populations from native and invasive ranges
Source: PeerJ. 2017 Aug 14;5:e3686. doi: 10.7717/peerj.3686 (PMC5560238; doi:10.7717/peerj.3686)
Supplement: Table S3 — * See Table 1 for explanation of the population code. [file peerj-05-3686-s004.docx]

| No | Pyrrolizidine alkaloid | Native populations * | | | | | | | Invasive populations * | | | | | |
| --- | --- | --- | --- | --- | --- | --- | --- | --- | --- | --- | --- | --- | --- | --- |
|  |  | Barcelona  （n=5） | Fribourg  ( n=5 ) | Óbidos  ( n=5 ) | Potsdam  ( n=5 ) | Puławy  ( n=5 ) | StAndrews ( n=5 ) | Dali.sts ( n=5 ) | | Dl.hsj ( n=5 ) | Lj.lsh  ( n=5 ) | Lj.xyl ( n=4 ) | Slj.djh ( n=5 ) | Slj.myz ( n=5 ) |
| 1 | senecionine | 28±25 | 40±26 | 47±27 | 25±24 | 20±16 | 32±17 | 34±22 | | 24±15 | 32±29 | 53±35 | 22±24 | 13.5±13.6 |
| 2 | senecionine N-oxide | 205±227 | 485±514 | 452±295 | 217±194 | 170±161 | 41±263 | 347±200 | | 309±291 | 244±184 | 529±230 | 102±99 | 57±41 |
| 3 | integerrimine | 3.8±3.6 | 5.3±3.8 | 5.8±3.7 | 4.2±3.4 | 4.7±3.4 | 6.5±4.8 | 4.9±3.6 | | 5.7±4.4 | 3.8±3.0 | 10.6±6.5 | 3.2±3.5 | 1.7±1.9 |
| 4 | integerrimine N-oxide | 37±42 | 87±90 | 66±42 | 44±37 | 42±34 | 98±76 | 66±41 | | 93±93 | 36±29 | 123±58 | 14.3±15.2 | 7.8±5.0 |
| 5 | senecivernine | 0.5±1.1 | <LOD | 1.1±0.8 | <LOD | <LOD | 2.1±1.2 | <LOD | | <LOD | 0.1±0.3 | <LOD | 0.3±0.6 | <LOD |
| 6 | retrorsine | 1.2±1.2 | 2.5±1.8 | 5.5±4.4 | 16±27 | 1.2±0.6 | 0.9±0.5 | 0.2±0.3 | | 0.4±0.5 | 1.3±0.7 | 1.3±0.6 | 1.2±1.4 | 2.0±2.7 |
| 7 | retrorsine N-oxide | 12±14 | 28±17 | 65±37 | 162±236 | 14±12 | 19.0±9.6 | 6.5±7.0 | | 4.5±3.0 | 15±12 | 17.4±8.9 | 9.6±8.6 | 17±23 |
| 8 | seneciphylline | 28±37 | 20±16 | 20±13 | 9.5±7.1 | 15±13 | 30±19 | 13.4±9.3 | | 19±14 | 16±14 | 11.5±8.3 | 12±14 | 7.7±7.7 |
| 9 | seneciphylline N-oxide | 281±437 | 223±270 | 187±110 | 86±77 | 126±107 | 343±226 | 123±75 | | 222±207 | 125±104 | 117±67 | 43±47 | 28±19 |
| 10 | spartioidine | 2.3±2.9 | 2.2±1.6 | 2.4±1.6 | 1.8±1.4 | 4.5±3.5 | 7.7±5.5 | 2.2±1.4 | | 4.8±3.8 | 1.8±1.3 | 2.4±1.3 | 1.0±1.2 | 0.8±0.7 |
| 11 | spartioidine N-oxide | 27±39 | 33±38 | 20±11 | 17±18 | 34±26 | 96±68 | 22±12 | | 59±56 | 12.4±10.0 | 22.1±8.6 | 5.7±6.3 | 3.4±2.0 |
| 12 | riddelliine N-oxide | 1.0±1.0 | 1.0±0.7 | 2.5±2.0 | 10.9±19.7 | 1.2±0.8 | 1.6±1.0 | <LOD | | 0.1±0.2 | 1.1±1.2 | 0.1±0.3 | 0.3±0.4 | 1.1±2.0 |
| 13 | unknown N-oxide 1 | <LOD | 0.8±1.2 | 0.1±0.2 | 0.9±1.8 | 0.8±1.2 | 6.0±6.1 | 0.6±1.1 | | 1.3±2.3 | 0.5±0.6 | 0.3±0.4 | 0.1±0.3 | <LOD |
| 14 | unknown N-oxide 2 | 1.3±2.1 | 6.3±10.6 | 2.7±2.3 | 2.5±5.2 | 2.4±3.1 | 13.8±12.4 | 3.4±3.6 | | 4.3±4.7 | 2.9±3.5 | 0.8±0.3 | 0.3±0.6 | 0.6±0.6 |
| 15 | unknown N-oxide 3 | 1.2±1.2 | 2.6±2.4 | 2.5±1.4 | 1.9±1.5 | 0.9±0.5 | 2.2±0.9 | 1.8±1.4 | | 2.1±2.0 | 1.0±1.2 | 2.5±0.5 | 0.4±0.5 | 0.4±0.4 |
| 16 | unknown N-oxide 4 | 16±20 | 32±21 | 23±11 | 18±18 | 21±14 | 100±34 | 34±21 | | 50±29 | 18.6±9.2 | 29.8±8.9 | 7.1±5.8 | 10.1±5.2 |
| 17 | unknown N-oxide 5 | 56±68 | 111±89 | 73±33 | 45±42 | 44±35 | 156±52 | 78±45 | | 80±57 | 63±37 | 55±12 | 28±24 | 29±20 |
| 18 | unknown N-oxide 6 | 0.9±0.8 | 3.5±3.3 | 3.4±1.5 | 3.0±2.7 | 4.3±2.8 | 8.6±6.8 | 5.3±4.1 | | 2.4±1.7 | 1.0±0.9 | 2.5±0.8 | 0.9±0.9 | 1.7±1.5 |
| 19 | unknown N-oxide 7 | 2.3±3.6 | 3.4±3.3 | 1.5±0.9 | 2.0±2.7 | 3.7±2.9 | 23.1±11.0 | 2.5±1.5 | | 6.7±4.7 | 1.9±1.0 | 2.0±0.9 | 0.9±0.9 | 1.1±1.0 |
| 20 | unknown N-oxide 8 | 11.5±16.1 | 10.0±10.4 | 7.9±5.6 | 3.3±4.0 | 6.1±4.0 | 24.9±10.9 | 5.3±2.7 | | 10.4±8.2 | 6.0±3.2 | 2.1±1.7 | 4.1±4.4 | 4.9±5.1 |
| Total PA concentration | | 716±919 | 1097±1081 | 989±538 | 674±513 | 516±411 | 1384±599 | 750±426 | | 899±775 | 583±417 | 982±380 | 255±254 | 187±142 |
